# Automated Patient-level Prostate Cancer Detection with Quantitative Diffusion Magnetic Resonance Imaging

**DOI:** 10.1016/j.euros.2022.11.009

**Published:** 2022-12-15

**Authors:** Allison Y. Zhong, Leonardino A. Digma, Troy Hussain, Christine H. Feng, Christopher C. Conlin, Karen Tye, Asona J. Lui, Maren M.S. Andreassen, Ana E. Rodríguez-Soto, Roshan Karunamuni, Joshua Kuperman, Christopher J. Kane, Rebecca Rakow-Penner, Michael E. Hahn, Anders M. Dale, Tyler M. Seibert

**Affiliations:** a Department of Radiation Medicine and Applied Sciences, University of California San Diego, La Jolla, CA, USA; b Department of Radiology, University of California San Diego, La Jolla, CA, USA; c Department of Circulation and Medical Imaging, Norwegian University of Science and Technology, Trondheim, Norway; d Department of Urology, University of California San Diego, La Jolla, CA, USA; e Department of Neurosciences, University of California San Diego, La Jolla, CA, USA; f Department of Bioengineering, University of California San Diego, La Jolla, CA, USA

**Keywords:** Cancer, Diffusion magnetic resonance imaging, Prostate, Quantitative magnetic resonance imaging, Restriction spectrum imaging

## Abstract

**Background:**

Multiparametric magnetic resonance imaging (mpMRI) improves detection of clinically significant prostate cancer (csPCa), but the subjective Prostate Imaging Reporting and Data System (PI-RADS) system and quantitative apparent diffusion coefficient (ADC) are inconsistent. Restriction spectrum imaging (RSI) is an advanced diffusion-weighted MRI technique that yields a quantitative imaging biomarker for csPCa called the RSI restriction score (RSI_rs_).

**Objective:**

To evaluate RSI_rs_ for automated patient-level detection of csPCa.

**Design, setting, and participants:**

We retrospectively studied all patients (*n* = 151) who underwent 3 T mpMRI and RSI (a 2-min sequence on a clinical scanner) for suspected prostate cancer at University of California San Diego during 2017–2019 and had prostate biopsy within 180 d of MRI.

**Intervention:**

We calculated the maximum RSI_rs_ and minimum ADC within the prostate, and obtained PI-RADS v2.1 from medical records.

**Outcome measurements and statistical analysis:**

We compared the performance of RSI_rs_, ADC, and PI-RADS for the detection of csPCa (grade group ≥2) on the best available histopathology (biopsy or prostatectomy) using the area under the curve (AUC) with two-tailed *α* = 0.05. We also explored whether the combination of PI-RADS and RSI_rs_ might be superior to PI-RADS alone and performed subset analyses within the peripheral and transition zones.

**Results and limitations:**

AUC values for ADC, RSI_rs_, and PI-RADS were 0.48 (95% confidence interval: 0.39, 0.58), 0.78 (0.70, 0.85), and 0.77 (0.70, 0.84), respectively. RSI_rs_ and PI-RADS were each superior to ADC for patient-level detection of csPCa (*p* < 0.0001). RSI_rs_ alone was comparable with PI-RADS (*p* = 0.8). The combination of PI-RADS and RSI_rs_ had an AUC of 0.85 (0.78, 0.91) and was superior to either PI-RADS or RSI_rs_ alone (*p* < 0.05). Similar patterns were seen in the peripheral and transition zones.

**Conclusions:**

RSI_rs_ is a promising quantitative marker for patient-level csPCa detection, warranting a prospective study.

**Patient summary:**

We evaluated a rapid, advanced prostate magnetic resonance imaging technique called restriction spectrum imaging to see whether it could give an automated score that predicted the presence of clinically significant prostate cancer. The automated score worked about as well as expert radiologists’ interpretation. The combination of the radiologists’ scores and automated score might be better than either alone.

## Take Home Message

An automated, quantitative biomarker from prostate magnetic resonance imaging—derived from a 2-min scan on a standard clinical scanner—was superior to conventional apparent diffusion coefficient and comparable with expert-determined Prostate Imaging Reporting and Data System for patient-level detection of clinically significant prostate cancer.

## 1 Introduction

Multiparametric magnetic resonance imaging (mpMRI) has become an integral part of prostate cancer (PCa) detection because it improves the detection of clinically significant prostate cancer (csPCa), while reducing the detection of indolent tumors [1], [2]. The standardized qualitative scoring system for mpMRI, Prostate Imaging Reporting and Data System (PI-RADS), has contributed to this success [3]. However, concerns remain regarding variable interpretation of mpMRI across readers, particularly when a PI-RADS ≥3 lesion is detected on mpMRI [4], [5], [6], [7]. This contributes to health disparities, as high-quality MRI is available only to a fraction of the men screened or diagnosed each year (22 million and >250 000, respectively, in the USA alone) [8], [9], [10], [11]. Quantitative mpMRI metrics are a promising means to standardize interpretation [12], [13], [14], [15], [16].

**Fig. 1:**
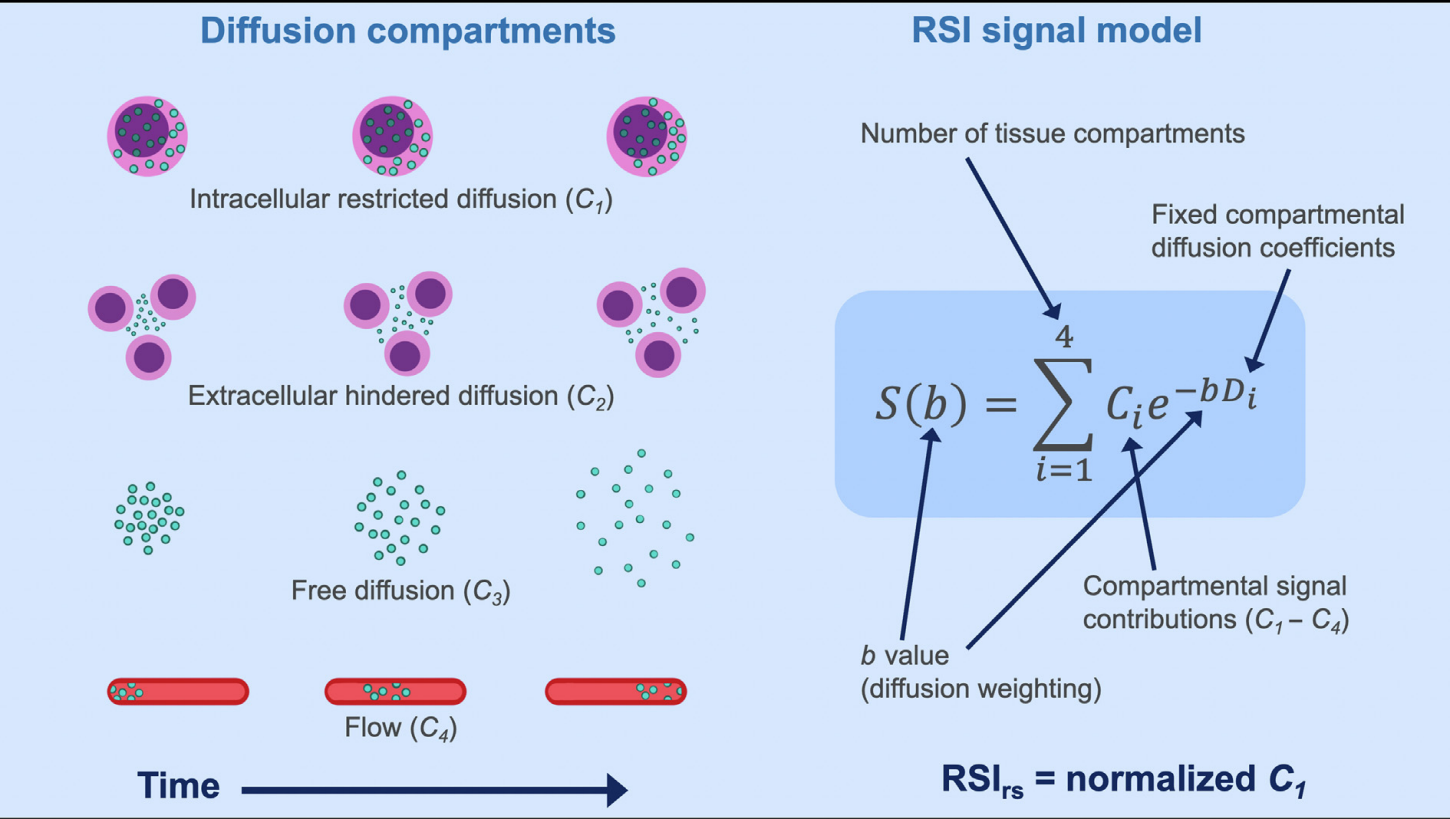
Diagram of a previously developed and validated four-compartment diffusion MRI model based on the restriction spectrum imaging (RSI) framework. The RSI restriction score (RSI_rs_) is a continuous cancer biomarker and is indicative of the contribution of intracellular restricted diffusion at a given location. MRI = magnetic resonance imaging. Digital artwork by Lia Seibert.

Restriction spectrum imaging (RSI) is an advanced technique for diffusion-weighted imaging (DWI) that accounts for a complex tissue microstructure by estimating the contributions of distinct tissue compartments believed to correspond to restricted intracellular water, hindered extracellular water, freely diffusing water, and vascular flow [15], [17]. We have recently developed a PCa MRI biomarker, called the RSI restriction score (RSI_rs_), which relies specifically on the restricted intracellular water signal (Fig. 1). RSI_rs_ gives improved cancer conspicuity and voxel-level PCa detection compared with the current clinical standard for quantitative DWI, the apparent diffusion coefficient (ADC) [17], [18].

The most important current clinical use of mpMRI is to guide the decision of whether to biopsy—that is, patient-level detection of csPCa [1], [2], [19], [20], [21], [22]. Here, we evaluate RSI_rs_ as a quantitative marker for patient-level detection of csPCa (grade group ≥2) without reliance on the subjective expert manual identification of specific lesions. We compared the performance of RSI_rs_ with that of conventional ADC as well as of PI-RADS v2.1 in a dataset not used in prior studies. We hypothesized that RSI_rs_ is superior to ADC for patient-level detection of csPCa on biopsy.

## 2 Patients and methods

### 2.1 Study population

With IRB approval, we retrospectively studied all men who underwent MRI with RSI for suspected PCa at University of California San Diego (UC San Diego) between 2017 and 2019, and had a prostate biopsy within 180 d of MRI. MRI examinations included standard mpMRI as well as a 2-min RSI series with four *b* values in a single acquisition (part of routine clinical prostate MRI at UC San Diego for use with an Food and Drug Administration–cleared postprocessing workflow).

### 2.2 MRI acquisition and processing

Scans were collected on a 3-T clinical MRI scanner (Discovery MR750; GE Healthcare, Waukesha, WI, USA) using a 32-channel phased-array body coil (acquisition parameters are shown in Table 1).

**Table 1:** Acquisition parameters for clinical multiparametric MRI and RSI

Sequence	FOV (mm^2^)	Voxel size (mm^3^)	Echo time (ms)	Repetition time (ms)	*b* values	Diffusion directions
RSI	240 × 120	2.5 × 2.5 × 6.0	68	4500	0, 500, 1000, 2000	2, 6, 6, 12^a^
Axial DWI 1	280 × 280	1.75 × 1.75 × 5.0	64	7990	0, 1000	1, 30^b^
Axial DWI 2	240 × 144	1.5 × 1.5 × 5.0	63	4000	0, 1400	1, 30^b^
Axial *T_2_* FSE	240 × 240	0.75 × 0.75 × 3.0	102	6080	NA	NA
Coronal *T_2_* FRFSE	200 × 200	0.52 × 0.89 × 3.0	102	4950	NA	NA
Axial *T_1_* LAVA-Flex	340 × 272	1.06 × 1.21 × 4.0	2	4	NA	NA
Sagittal *T_2_* FRFSE	250 × 250	0.65 × 0.71 × 4.0	103	3690	NA	NA
DCE	240 × 240	0.94 × 1.43 × 3.0	2	4	NA	NA

DCE = dynamic contrast enhanced; DWI = diffusion-weighted imaging; FOV = field of view; FSE = fast spin echo; FRFSE = fast recovery fast spin echo; LAVA = liver acquisition with volume acquisition; MRI = magnetic resonance imaging; NA = not available; RSI = restriction spectrum imaging.

a Default tensor directions (six directions, NEX = 2).

b Default orthogonal directions (three directions, NEX = 10).

We performed postprocessing of MRI data in MATLAB (MathWorks, Natick, MA, USA), including corrections for distortions from *B_0_* inhomogeneity, gradient nonlinearity, and eddy currents [23], [24]. We performed RSI calculations as described previously (Fig. 1) [17], [18]. Briefly, we corrected diffusion signals for noise and distortion, and then scaled by the median *b* = 0 signal within each patient’s prostate. We modeled signal intensity for each *b* value as a linear combination of exponential decays representing four diffusion compartments with previously empirically determined diffusion coefficients: 1.0e−4, 1.8e−3, 3.6e−3, and >>3.0e−3 mm^2^/s, approximately representing restricted, hindered, free diffusion, and flow, respectively [17]. All postprocessing was accomplished on a desktop computer (64 GB RAM; 8-core, 2.40 GHz Intel Xeon E5-2630 v3 CPU).

ADC maps were generated automatically, per clinical routine, using vendor software on the MRI system and the Axial DWI acquisition with *b* values of 0 and 1000 s/mm^2^. As a secondary analysis, we also calculated voxel-wise ADC from the RSI acquisition in MATLAB, using *b* values of 0, 500, and 1000 s/mm^2^, and repeated the main analyses using these alternate ADC maps.

We manually segmented the prostate gland, peripheral zone, and central gland on T_2_-weighted imaging and verified on DWI volumes using MIM (MIM Software Inc., Cleveland, OH, USA). We visually inspected these imaging volumes to ensure that there was no severe distortion or movement. More variability might be expected in routine clinical use, where these careful quality assurance steps might not be feasible, so we added a uniform 5-mm margin on the prostate contour to allow for the possibility of imperfections in the contour or modest movement effects.

### 2.3 Clinical data

We reviewed clinical records to obtain histopathology results (highest grade group [25] on biopsy or prostatectomy, if applicable) and imaging results (highest PI-RADS category reported). MRI examinations were read per routine practice by board-certified, subspecialty fellowship–trained radiologists, using all available images and standard PI-RADS criteria. Transition from PI-RADS v2 to v2.1 occurred in 2019; v2 results were updated retrospectively to v2.1 by a board-certified radiologist with 7 yr of experience (M.E.H.) [3], [26]. Images from the MRI acquisition were also available to the radiologists at the time of interpretation, with postprocessing applied via commercially available software per clinical routine. After providing standard PI-RADS categories based on conventional images, radiologists could comment on the MRI images in their report. However, the quantitative RSI_rs_ biomarker evaluated in this study was not available at the time of clinical interpretation. Prostate biopsies (systematic, targeted, or both) and radical prostatectomies (where applicable) were performed per clinical routine. Board-certified pathologists interpreted histopathology specimens from biopsy and prostatectomy specimens. Thus, both clinical imaging and pathology results in this study represent real-world performance at an academic medical center.

### 2.4 Statistical analyses

#### 2.4.1 Primary analysis: patient-level detection of csPCa

We generated receiver operating characteristic (ROC) curves for patient-level detection of csPCa using ADC, RSI_rs_, and PI-RADS. In the primary analysis, we analyzed RSI_rs_ and ADC as quantitative metrics, taking the maximum RSI_rs_ and minimum ADC within the prostate. It is important to note that the use of the minimum ADC here differs from clinical practice, where an expert radiologist typically identifies a suspicious lesion and then calculates the mean ADC from all or part of that lesion [27]. Our approach using the maximum RSI_rs_ and minimum ADC is analogous to the use of the maximum standardized uptake value in positron emission tomography imaging for cancer [28]. We chose this prostate-wide approach to evaluate whether a quantitative metric could be used in fully automated fashion within the prostate, without relying on subjective delineation of individual lesions that depend on reader experience [11]. We considered biopsies finding only grade group 1 cancers (Gleason ≤6) or benign tissue as negative results for the ROC curves. We assessed performance by the area under the ROC curve (AUC) and made statistical comparisons via 10 000 bootstrap samples to calculate 95% confidence intervals and bootstrap *p* values for the difference between the performance (AUC) of ADC, RSI_rs_, and PI-RADS [29]. We used two-sided *α* = 0.05 to determine statistical significance.

We used procedures analogous to those described above for subsequent analyses as follows:

#### 2.4.2 Quantitative diffusion MRI within PI-RADS categories

To determine whether RSI_rs_ enhances the detection of higher-grade PCa compared with PI-RADS alone, we repeated the RSI_rs_ patient-level analysis within the strata of each PI-RADS category (ie, 3, 4, and 5).

#### 2.4.3 Combination of PI-RADS and RSI

To explore overall performance of the combination of PI-RADS and RSI_rs_, we generated an ROC curve for PI-RADS + RSI_rs_ by concatenating the within–PI-RADS strata performance from above (ie, the logistic posterior probabilities) across categories. We then calculated the AUC of the resulting ROC curve for PI-RADS + RSI_rs_ and compared it with either PI-RADS or RSI_rs_ alone.

#### 2.4.4 Peripheral zone and transition zone

We again repeated the patient-level analysis in subgroups with lesions in only either the peripheral zone or the transition zone. For the transition zone analysis, we limited the search for the maximum RSI_rs_ and minimum ADC to the central gland (transition and central zones). We performed an analogous analysis for patients with peripheral zone cancers. Then, to evaluate whether zone-specific searching was necessary to optimize performance, we repeated the transition zone and peripheral zone subgroup analyses but allowed the search for the maximum RSI_rs_ and minimum ADC to include the whole prostate.

## 3 Results

A total of 151 patients met the criteria for inclusion (characteristics are summarized in Table 2). Ten radiologists had interpreted the imaging for these 151 patients, reading a median of 18 cases each (interquartile range [IQR]: four to 24 cases). The radiologists were board certified and subspecialty fellowship trained, with a median of 4 yr of experience (IQR: 4–9 yr). More experienced radiologists read more cases, so the mean number of years of experience per case was 8.5 yr (standard deviation: 1 yr).

**Table 2:** Characteristics of the patients included in this study

Age (yr), median (IQR)			66 (59–72)
Time from MRI to biopsy (d), median (IQR)			16 (1–35)
PSA at time of MRI (ng/ml), median (IQR)			7.3 (5.3–10.4)
Prostate volume (ml), median (IQR)			45 (34–61)
PSA density (ng/ml^2^), median (IQR)			0.16 (0.11–0.25)
Previous biopsy	Biopsy naïve		105
	Had undergone past biopsy		46
	Past biopsy benign		12
	Past biopsy grade group 1		29
	Past biopsy grade group 2		3
	Past biopsy grade group 3		2
PI-RADS version	v2		104
	v2.1		47
Best available pathology	Systematic		7
	Targeted		17
	Systematic and targeted		85
	Prostatectomy		42
Clinical T stage	Negative biopsy		25
	T1c		94
	T2a		13
	T2b		11
	T2c		8
		Benign or low-grade PCa	csPCa
PI-RADS category (% of detection rate)	1	0	0
	2	2 (40.0%)	3 (60.0%)
	3	23 (85.2%)	4 (14.8%)
	4	30 (54.5%)	25 (45.5%)
	5	10 (15.6%)	54 (84.4%)
Gleason grade group	None	25	
	1	40	
	2		38
	3		20
	4		16
	5		12

csPCa = clinically significant PCa; IQR = interquartile range; MRI = magnetic resonance imaging; PCa = prostate cancer; PSA = prostate-specific antigen.

### 3.1 Primary analysis: patient-level detection of csPCa

**Fig. 2:**
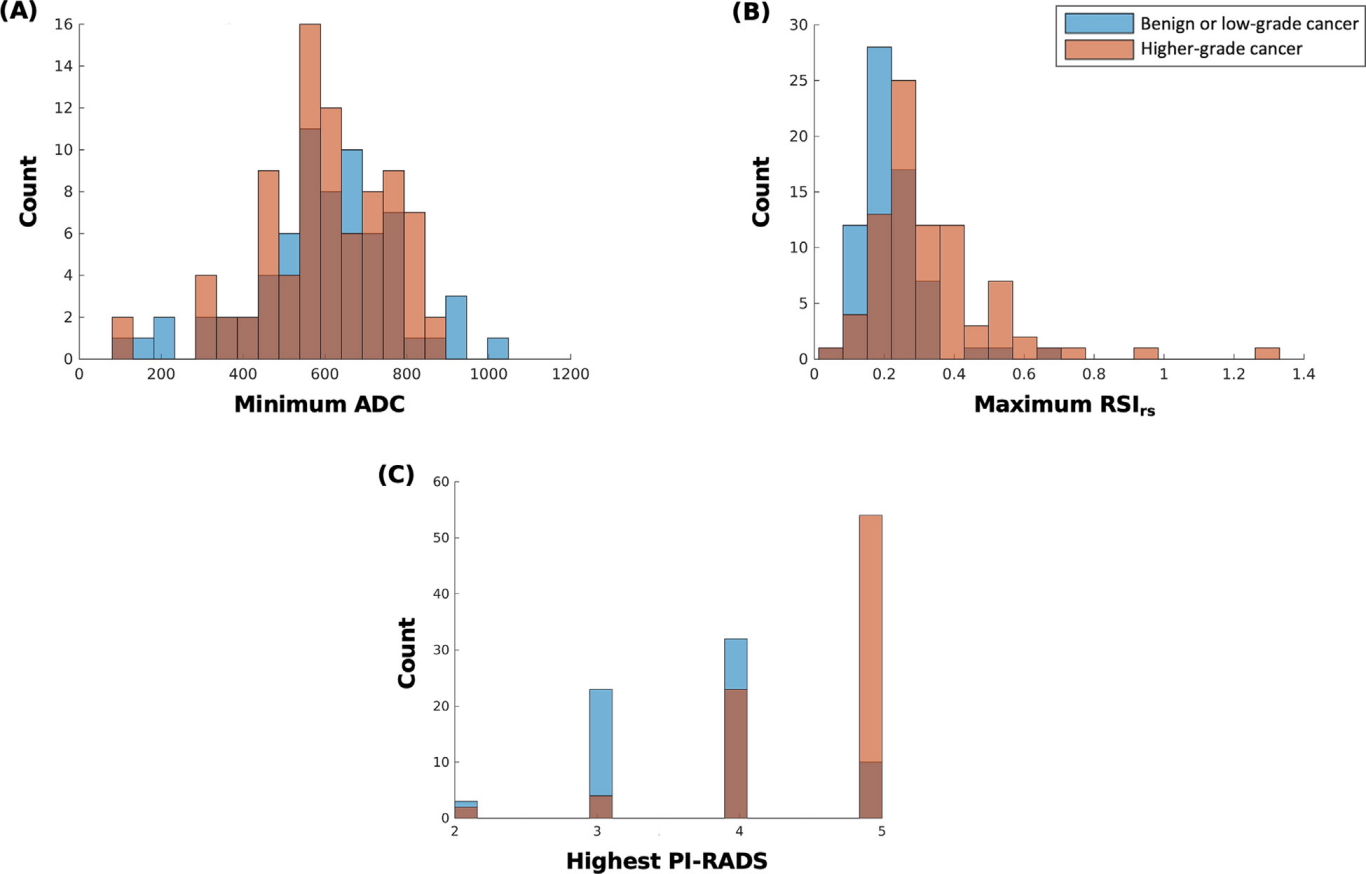
Histograms of (A) minimum conventional ADC in the prostate, (B) maximum RSI_rs_ in the prostate, and (C) highest PI-RADS v2.1 category in the prostate. The blue color represents patients with no cancer or low-grade cancer, orange represents patients with higher-grade (grade group ≥2) prostate cancer, and brown represents overlap of blue and orange. ADC = apparent diffusion coefficient; PI-RADS = Prostate Imaging Reporting and Data System; RSI_rs_ = restriction spectrum imaging restriction score.

**Fig. 3:**
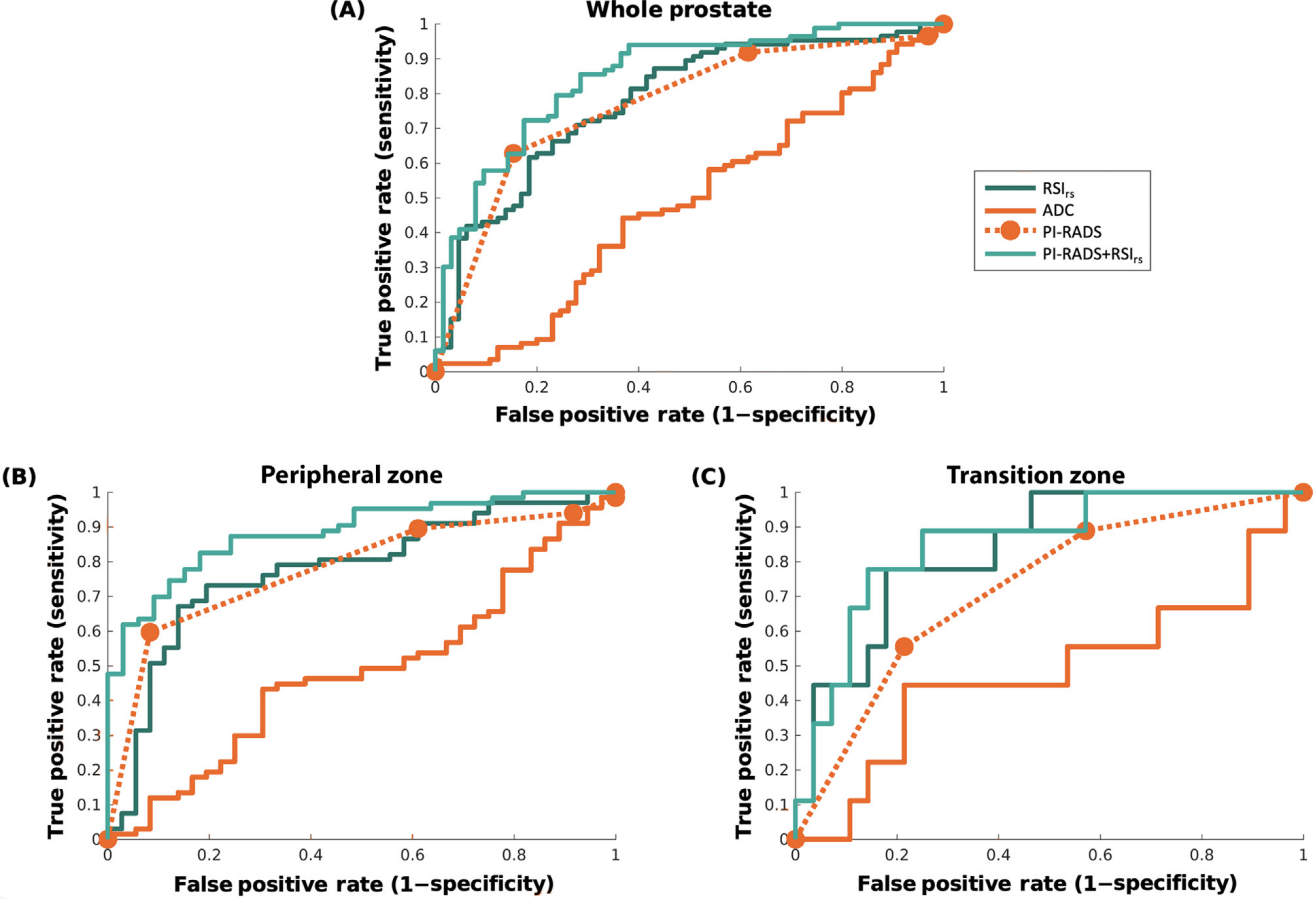
Receiver operator characteristic (ROC) curves for conventional ADC (solid orange), RSI_rs_ (dark green), PI-RADS (dashed orange), and the combination of PI-RADS and RSI_rs_ (PI-RADS + RSI_rs_, light green) for patient-level detection of higher-grade prostate cancer (A) anywhere in the prostate, (B) in the peripheral zone, and (C) in the transition zone. For the whole prostate, the AUC values for ADC, RSI_rs_, PI-RADS, and PI-RADS + RSI_rs_ were 0.48 (95% confidence interval: 0.39, 0.58), 0.78 (0.70, 0.85), 0.77 (0.70, 0.84), and 0.85 (0.78, 0.91), respectively. For peripheral zone cases, the AUC values for ADC, RSI_rs_, PI-RADS, and PI-RADS + RSI_rs_ were 0.48 (0.37, 0.60), 0.78 (0.68, 0.87), 0.78 (0.69, 0.86), and 0.89 (0.82, 0.95), respectively. For transition zone cases, the AUC values for ADC, RSI_rs_, PI-RADS, and PI-RADS + RSI_rs_ were 0.48 (0.23, 0.72), 0.84 (0.68, 0.95), 0.73 (0.54, 0.88), and 0.86 (0.70, 0.97), respectively. ADC = apparent diffusion coefficient; AUC = area under the curve; PI-RADS = Prostate Imaging Reporting and Data System; RSI_rs_ = restriction spectrum imaging restriction score.

All 151 patients were included in the primary (whole-prostate) analysis. AUC values for ADC, RSI_rs_, and PI-RADS are reported in Table 3. Both RSI_rs_ (*p* < 0.0001) and PI-RADS (*p* < 0.0001) were superior to ADC as a patient-level classifier of higher-grade PCa. The performance of RSI_rs_ was comparable with that of PI-RADS (*p* = 0.8). The histograms and ROC curves for the primary analysis are shown in Fig. 2, Fig. 3A, respectively.

**Table 3:** Comparison of patient-level performance for the detection of grade group ≥2 cancer between minimum ADC, maximum RSI_rs_, highest PI-RADS, and within–PI-RADS maximum RSI_rs_ (PI-RADS + RSI_rs_)

Analysis	ADC	RSI_rs_	PI-RADS	PI-RADS + RSI_rs_
Whole prostate (*n* = 151)	0.48	0.78^a^	0.77	0.85^b^
	(0.39, 0.58)	(0.70, 0.85)	(0.70, 0.84)	(0.78, 0.91)
Peripheral zone (*n* = 103)	0.48	0.78^a^	0.78	0.89^b^
	(0.37, 0.60)	(0.68, 0.87)	(0.69, 0.86)	(0.82, 0.95)
Transition zone (*n* = 37)	0.48	0.84^a^	0.73	0.86^b^
	(0.23, 0.72)	(0.68, 0.95)	(0.54, 0.88)	(0.70, 0.97)
PI-RADS 3 (*n* = 27)	0.46	0.70	0.50	–
	(0.26,0.66)	(0.50,0.87)	–	–
PI-RADS 4 (*n* = 55)	0.33	0.74^a^	0.50	–
	(0.19,0.47)	(0.60,0.87)	–	–
PI-RADS 5 (*n* = 64)	0.56	0.73	0.50	–
	(0.33,0.77)	(0.54,0.88)	–	–

ADC = apparent diffusion coefficient; RSI_rs_ = restriction spectrum imaging restriction score; PI-RADS = Prostate Imaging Reporting and Data System.

Numbers shown are area under the receiver operating characteristic curve and 95% confidence intervals.

a RSI_rs_ was superior to ADC.

b PI-RADS + RSI_rs_ was superior to PI-RADS alone (*p* < 0.05).

### 3.2 Quantitative diffusion MRI within PI-RADS categories

We found 27, 55, and 64 patients with the maximum PI-RADS categories 3, 4, and 5, respectively. AUC values for the maximum prostate RSI_rs_ within the PI-RADS groups 3, 4, and 5 are shown in Table 3. Performance for RSI_rs_ was numerically greater than that for ADC in each subset, although confidence intervals were wide. The difference was statistically significant within patients with PI-RADS 4 lesions (*p* < 0.0001) but not within patients with PI-RADS 3 (*p* = 0.10) or 5 (*p* = 0.13) lesions.

There was no significant difference in performance between the alternate ADC maps and vendor-calculated ADC maps (*p* = 0.24).

### 3.3 Combination of PI-RADS and RSI

AUC values for RSI_rs_ concatenated within–PI-RADS subsets (PI-RADS + RSI_rs_) and applied to all 151 patients are shown in Table 3. PI-RADS + RSI_rs_ was superior to either PI-RADS (*p* = 0.001) or RSI_rs_ (*p* = 0.03) alone. ROC curves are shown in Figure 3A.

### 3.4 Peripheral zone

We found 103 patients with a peripheral zone lesion and no transition zone lesion (15 benign, 23 grade group 1, and 65 csPCa). AUC values are shown in Table 3. RSI_rs_ performance was comparable with that of PI-RADS for the peripheral zone (*p* = 0.98) and superior to that of ADC (*p* = 0.0002). ROC curves are shown in Figure 2B. PI-RADS + RSI_rs_ was superior to either PI-RADS (*p* = 0.005) or RSI_rs_ alone (*p* = 0.003). Similar results were obtained when searching the whole prostate for the maximum RSI_rs_.

### 3.5 Transition zone

**Fig. 4:**
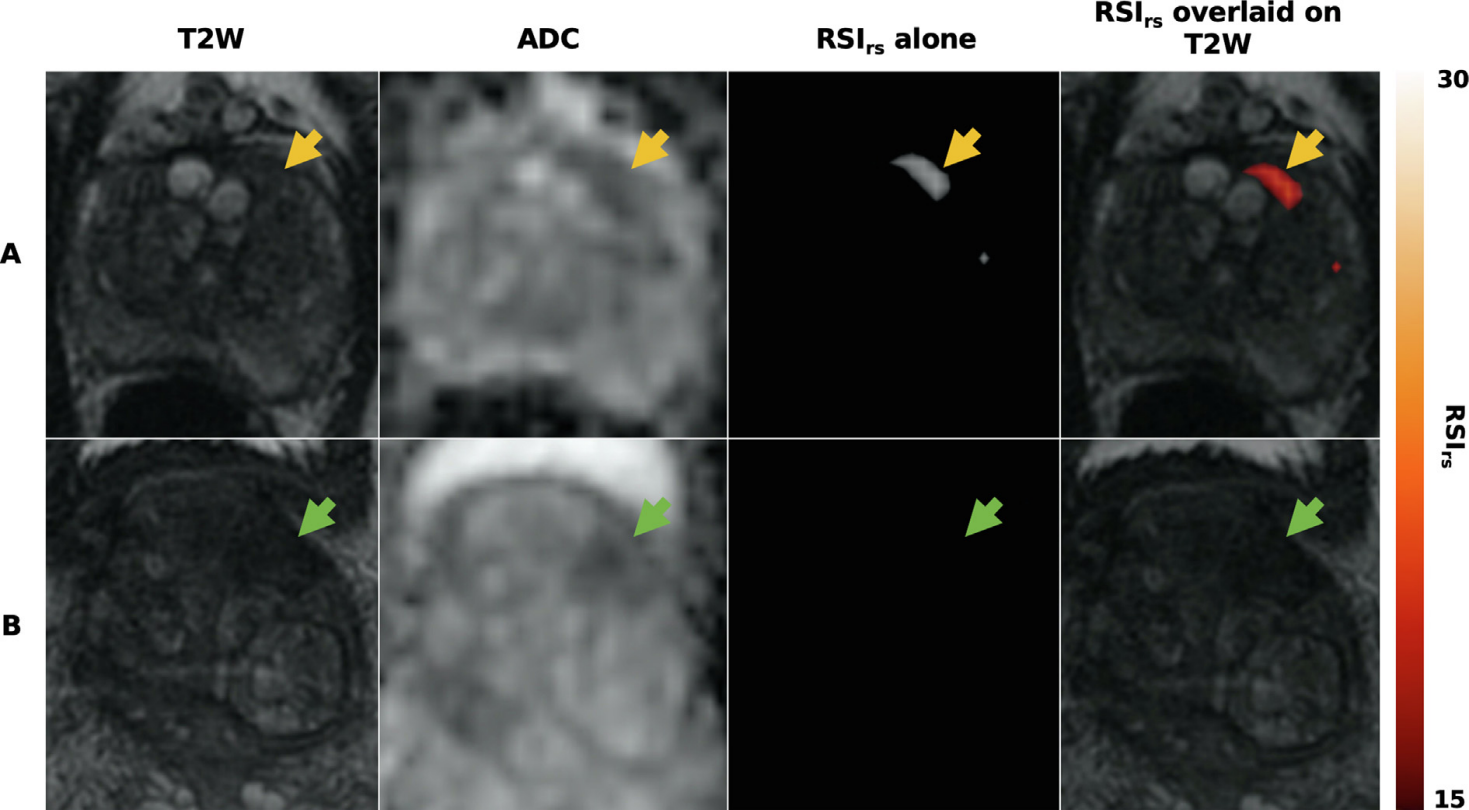
Axial images from two patients with transition zone lesions: T_2_-weighted MRI (T2W), conventional ADC, and RSI_rs_. Patient A had a PI-RADS 3 lesion (yellow arrow) in the left transition zone; he underwent prostatectomy and was found to have Gleason 3 + 4 prostate cancer. Patient B had a PI-RADS 5 lesion (green arrow) on multiparametric MRI, with subsequent biopsy showing benign prostatic tissue with acute and chronic inflammation. The RSI_rs_ map readily highlights the cancer for patient A. The RSI_rs_ map for patient B has no false-positive voxels (and is shown on the same color scale as the map for patient A). ADC = apparent diffusion coefficient; MRI = magnetic resonance imaging; PI-RADS = Prostate Imaging Reporting and Data System; RSI_rs_ = restriction spectrum imaging restriction score.

We found 37 patients with a transition zone lesion and no peripheral zone lesion (14 benign, 15 grade group 1, and eight csPCa). AUC values are shown in Table 3. RSI_rs_ performance was superior to that of ADC (*p* < 0.0001) in the transition zone. RSI_rs_ performance was numerically superior to that of PI-RADS, but this difference was not statistically significant (*p* = 0.08). PI-RADS + RSI_rs_ was superior to PI-RADS (*p* = 0.005) but not RSI_rs_ alone (*p* = 0.63). ROC curves are shown in Figure 3C. RSI_rs_ images and ADC maps for two patients with transition zone lesions are shown in Figure 4. Similar results were obtained when searching the whole prostate for the maximum RSI_rs_, suggesting that zone-specific searching may not be necessary.

## 4 Discussion

RSI_rs_ performed well for quantitative, automated detection of csPCa at the patient level. ADC proved unreliable as a quantitative marker with an analogous approach. We note that routine clinical use of ADC is not automated and fully quantitative; rather, it is typically used within expert-defined lesions. RSI_rs_ was based solely on a 2-min diffusion MRI acquisition on a standard clinical scanner; yet, performance was comparable with PI-RADS categories assigned by experts using all images from a complete mpMRI examination.

An analysis of the transition zone was underpowered because relatively few csPCa cases could be included (there were many more false positives from PI-RADS interpretation than true positives in the transition zone). With that limitation, there was no suggestion of worse performance for RSI_rs_ in the transition zone, with an AUC of 0.84 (0.68, 0.95) for RSI_rs_, compared with 0.73 (0.54, 0.88) for PI-RADS (*p* = 0.08). This should be investigated further in larger datasets, as a prior retrospective analysis using a different RSI model found superior specificity for RSI in the transition zone [30].

In exploratory analyses, we found that combining PI-RADS categories and the maximum RSI_rs_ might improve performance over either of these alone. RSI_rs_ had an AUC of ≥0.70 within each PI-RADS subset, including PI-RADS 3. Concatenating the within–PI-RADS ROC results showed that the combination of PI-RADS and RSI_rs_ also performed better than PI-RADS alone across the full dataset. This last finding should be interpreted cautiously because there were relatively few patients in each PI-RADS category subset. In the future, larger datasets will permit development of a multivariable model with PI-RADS and RSI_rs_, which could then be validated in an independent dataset. In contrast, all other findings in this study already represent validation tests in an independent dataset from the one used to develop the quantitative RSI_rs_ biomarker.

Our approach is clinically feasible. RSI_rs_ was calculated from a 2-min acquisition on a standard clinical scanner, and all postprocessing was achieved in 14 min per patient using a desktop computer. Similar RSI models are already commercially available and in clinical use. The present study demonstrates performance of a quantitative RSI metric for csPCa detection in a completely independent dataset from that used to develop the model and with a distinct acquisition protocol (different *b* values and echo time).

PI-RADS categories for this study were assigned during routine clinical practice. All readers were board-certified and subspecialty-trained attending radiologists at an academic center and adhered to PI-RADS standards, but this does not preclude some inter-reader variability. The goal of this analysis was not to use idealized PI-RADS implementation with central reads, but rather to obtain a real-world comparator for the quantitative biomarker. Performance for PI-RADS here is within the range of expected values [4]. Clinical decision-making surrounding biopsy may have been influenced by any number of imaging and nonimaging clinical factors, per standard of care. However, as none of these additional risk factors are formally incorporated into PI-RADS, there is nothing to suggest that this decision-making would unduly influence the relative performance of PI-RADS, ADC, and RSI_rs_ among men who did undergo biopsy. Studies to incorporate RSI_rs_ and other clinical factors for optimal decision-making are ongoing and would only improve on the encouraging performance demonstrated in the present work.

Limitations of this study include its retrospective, single-institution design. Patients who did not undergo biopsy were excluded, although mpMRI is known to have a high negative predictive value, and the population included in this study is most likely to benefit from improvements in quantitative MRI. Imaging for this dataset was acquired on a single scanner. This study relied on PI-RADS interpretation per clinical routine, which reflects real-world practice at our institution but may differ from the centralized review by one or two readers. Biopsy as the gold standard is also a limitation (some cancers may be missed), although this also reflects real-world performance; neither prostatectomy nor template-mapping biopsy is offered for routine diagnosis. In a post hoc subset analysis, the main findings were unchanged when evaluating only those who did not have a prostatectomy (results not shown). We could not adequately evaluate lesion-level performance because the retrospective analysis does not permit histopathologic verification of lesions detected by RSI_rs_, although the patient-level decision of whether to biopsy is the most important clinical use case of MRI [1], [2], and voxel-level performance with RSI_rs_ was quite good in prior studies [17], [18].

## 5 Conclusions

In an independent validation, the performance achieved by RSI_rs_ for patient-level detection of csPCa was superior to that of conventional ADC and comparable with that of routine, clinical PI-RADS. The combination of PI-RADS and RSI_rs_ may perform better than either RSI_rs_ or PI-RADS alone. These patterns held true within the transition zone, a region known to be more challenging for standard mpMRI. RSI_rs_ holds promise as a quantitative marker and should prospectively be studied for improvement of PCa diagnosis.

  ***Author contributions*:** Tyler M. Seibert had full access to all the data in the study and takes responsibility for the integrity of the data and the accuracy of the data analysis.

*Study concept and design*: Zhong, Dale, Seibert.

*Acquisition of data*: Zhong, Digma, Hussain, Feng, Conlin, Tye, Kuperman, Kane, Rakow-Penner, Hahn, Dale, Seibert.

*Analysis and interpretation of data*: All authors.

*Drafting of the manuscript*: Zhong, Conlin, Seibert.

*Critical revision of the manuscript for important intellectual content*: All authors.

*Statistical analysis*: Zhong, Conlin, Dale, Seibert.

*Obtaining funding*: Dale, Seibert.

*Administrative, technical, or material support*: Hussain, Conlin, Kuperman, Dale.

*Supervision*: Dale, Seibert.

*Other*: None.

  ***Financial disclosures:*** Tyler M. Seibert certifies that all conflicts of interest, including specific financial interests and relationships and affiliations relevant to the subject matter or materials discussed in the manuscript (eg, employment/affiliation, grants or funding, consultancies, honoraria, stock ownership or options, expert testimony, royalties, or patents filed, received, or pending), are the following: Michael E. Hahn reports honoraria from Multimodal Imaging Services Corporation and research funding from General Electric Healthcare. Anders M. Dale is a founder of and holds equity in CorTechs Labs, Inc., and serves on its Scientific Advisory Board; he is a member of the Scientific Advisory Board of Human Longevity, Inc. and receives funding through research agreements with General Electric Healthcare. The terms of these arrangements have been reviewed and approved by the University of California San Diego in accordance with its conflict-of-interest policies. Tyler M. Seibert reports honoraria from Multimodal Imaging Services Corporation, Varian Medical Systems, and WebMD; he has an equity interest in CorTechs Labs, Inc. and also serves on its Scientific Advisory Board. These companies might potentially benefit from the research results. The terms of this arrangement have been reviewed and approved by the University of California San Diego in accordance with its conflict-of-interest policies.

  ***Funding/Support and role of the sponsor*:** This work was supported, in part, by the National Institutes of Health (NIH/NIBIB K08 EB026503), the American Society for Radiation Oncology, and the Prostate Cancer Foundation.

## References

[1] Moses K, Sprenkle P, Box G, et al. NCCN guidelines for prostate cancer early detection version 1.2022. 2022.

[2] Mottet N., van den Bergh R.C.N., Briers E. (2021). EAU-EANM-ESTRO-ESUR-SIOG guidelines on prostate cancer—2020 update. Part 1: screening, diagnosis, and local treatment with curative intent. Eur Urol.

[3] Turkbey B., Rosenkrantz A.B., Haider M.A. (2019). Prostate Imaging Reporting and Data System version 2.1: 2019 update of Prostate Imaging Reporting and Data System version 2. Eur Urol.

[4] Westphalen A.C., McCulloch C.E., Anaokar J.M. (2020). Variability of the positive predictive value of PI-RADS for prostate MRI across 26 centers: experience of the Society of Abdominal Radiology Prostate Cancer Disease-focused Panel. Radiology.

[5] Milot L. (2020). Variation of PI-RADS interpretations between experts: a significant limitation. Radiology.

[6] Turkbey B., Oto A. (2021). Factors impacting performance and reproducibility of PI-RADS. Can Assoc Radiol J.

[7] Sackett J., Shih J.H., Reese S.E. (2021). Quality of prostate MRI: is the PI-RADS standard sufficient?. Acad Radiol.

[8] Siegel R.L., Miller K.D., Fuchs H.E., Jemal A. (2022). Cancer statistics, 2022. CA Cancer J Clin.

[9] El Khoury C.J., Ros P.R. (2021). A systematic review for health disparities and inequities in multiparametric magnetic resonance imaging for prostate cancer diagnosis. Acad Radiol.

[10] Leapman M.S., Dinan M., Pasha S. (2022). Mediators of racial disparity in the use of prostate magnetic resonance imaging among patients with prostate cancer. JAMA Oncol.

[11] Penzkofer T., Padhani A.R., Turkbey B. (2021). ESUR/ESUI position paper: developing artificial intelligence for precision diagnosis of prostate cancer using magnetic resonance imaging. Eur Radiol.

[12] Shukla-Dave A., Obuchowski N.A., Chenevert T.L. (2019). Quantitative Imaging Biomarkers Alliance (QIBA) recommendations for improved precision of DWI and DCE-MRI derived biomarkers in multicenter oncology trials. J Magn Reson Imaging.

[13] Lee G.H., Chatterjee A., Karademir I. (2022). Comparing radiologist performance in diagnosing clinically significant prostate cancer with multiparametric versus hybrid multidimensional MRI. Radiology.

[14] Si Y., Liu R.-B. (2018). Diagnostic performance of monoexponential DWI versus diffusion kurtosis imaging in prostate cancer: a systematic review and meta-analysis. AJR Am J Roentgenol.

[15] Brunsing R.L., Schenker-Ahmed N.M., White N.S. (2017). Restriction spectrum imaging: an evolving imaging biomarker in prostate MRI. J Magn Reson Imaging.

[16] Johnston E.W., Bonet-Carne E., Ferizi U. (2019). VERDICT MRI for prostate cancer: intracellular volume fraction versus apparent diffusion coefficient. Radiology.

[17] Conlin C.C., Feng C.H., Rodriguez-Soto A.E. (2021). Improved characterization of diffusion in normal and cancerous prostate tissue through optimization of multicompartmental signal models. J Magn Reson Imaging.

[18] Feng C.H., Conlin C.C., Batra K. (2021). Voxel-level classification of prostate cancer using a four-compartment restriction spectrum imaging model. J Magn Reson Imaging.

[19] Ahmed H.U., El-Shater Bosaily A., Brown L.C. (2017). Diagnostic accuracy of multi-parametric MRI and TRUS biopsy in prostate cancer (PROMIS): a paired validating confirmatory study. Lancet.

[20] Kasivisvanathan V., Rannikko A.S., Borghi M. (2018). MRI-targeted or standard biopsy for prostate-cancer diagnosis. N Engl J Med.

[21] Klotz L., Chin J., Black P.C. (2021). Comparison of multiparametric magnetic resonance imaging–targeted biopsy with systematic transrectal ultrasonography biopsy for biopsy-naive men at risk for prostate cancer: a phase 3 randomized clinical trial. JAMA Oncol.

[22] Eklund M., Jäderling F., Discacciati A. (2021). MRI-targeted or standard biopsy in prostate cancer screening. N Engl J Med.

[23] White N.S., McDonald C., McDonald C.R. (2014). Diffusion-weighted imaging in cancer: physical foundations and applications of restriction spectrum imaging. Cancer Res.

[24] Holland D., Kuperman J.M., Dale A.M. (2010). Efficient correction of inhomogeneous static magnetic field-induced distortion in Echo Planar Imaging. NeuroImage.

[25] Egevad L., Delahunt B., Srigley J.R., Samaratunga H. (2016). International Society of Urological Pathology (ISUP) grading of prostate cancer—an ISUP consensus on contemporary grading. Acta Pathol Microbiol Immunol Scand.

[26] Weinreb J.C., Barentsz J.O., Choyke P.L. (2016). PI-RADS Prostate Imaging - Reporting and Data System: 2015, version 2. Eur Urol.

[27] Manetta R., Palumbo P., Gianneramo C. (2019). Correlation between ADC values and Gleason score in evaluation of prostate cancer: multicentre experience and review of the literature. Gland Surg.

[28] Barrington S.F., Qian W., Somer E.J. (2010). Concordance between four European centres of PET reporting criteria designed for use in multicentre trials in Hodgkin lymphoma. Eur J Nucl Med Mol Imaging.

[29] Efron B., Tibshirani R. (1986). Bootstrap methods for standard errors, confidence intervals, and other measures of statistical accuracy. Stat Sci.

[30] Felker E.R., Raman S.S., Lu D.S.K. (2019). Utility of multiparametric MRI for predicting residual clinically significant prostate cancer after focal laser ablation. Am J Roentgenol.

